# Inhibition of PTEN activity promotes IB4‐positive sensory neuronal axon growth

**DOI:** 10.1111/jcmm.15648

**Published:** 2020-08-03

**Authors:** Li‐Yu Zhou, Feng Han, Shi‐Bin Qi, Jin‐Jin Ma, Yan‐Xia Ma, Ji‐Le Xie, Hong‐Cheng Zhang, Xin‐Ya Fu, Jian‐Quan Chen, Bin Li, Hui‐Lin Yang, Feng Zhou, Saijilafu

**Affiliations:** ^1^ Department of Orthopaedics The First Affiliated Hospital of Soochow University Orthopedic Institute Soochow University Suzhou P. R. China

**Keywords:** axon regeneration, IB4‐positive neurons, PTEN, sensory neuron

## Abstract

Traumatic nerve injuries have become a common clinical problem, and axon regeneration is a critical process in the successful functional recovery of the injured nervous system. In this study, we found that peripheral axotomy reduces PTEN expression in adult sensory neurons; however, it did not alter the expression level of PTEN in IB4‐positive sensory neurons. Additionally, our results indicate that the artificial inhibition of PTEN markedly promotes adult sensory axon regeneration, including IB4‐positive neuronal axon growth. Thus, our results provide strong evidence that PTEN is a prominent repressor of adult sensory axon regeneration, especially in IB4‐positive neurons.

## 1 INTRODUCTION

Functional recovery after nerve injury is often unsatisfactory as many patients suffer from permanent complications, such as paralysis. Axons are the main transmission pathway of electric impulses between a neuron and its downstream effector. Thus, axon regeneration is a critical determinant for the successful functional recovery of the injured nervous system. To date, recent studies have identified that the PTEN gene is a critical regulator of the intrinsic axon regeneration ability of mature neurons and that PTEN knockout significantly promotes axon regeneration in both central nervous system and peripheral nervous system.^1^, ^2^, ^3^

It has been reported that mammalian dorsal root ganglion neurons (DRG) can be divided into several subpopulations according to its Griffonia simplicifolia isolectin B4 (IB4) affinity.^4^, ^5^ Additionally, IB4‐labelled neurons (IB4^+^ ) are difficult to regenerate axon after injury, and even the forced overexpression of axon growth promoting molecules, such as α7β1‐integrin and GAP43, failed to enhance the axon growth of IB4^+^ neurons.^6^ These findings indicate that IB4^+^ neurons lack the intrinsic ability to regenerate axons following injury. Interestingly, a recent study found that IB4^+^ neurons usually have an elevated expression of PTEN protein.^7^ Therefore, we suggested that the PTEN gene is one of the crucial inhibitors of IB4^+^ neuronal axon regeneration. Thus, the suppression of PTEN activity may increase the intrinsic axon growth ability of IB4^+^ neurons and further promote its axon regeneration after injury.

## 2 METHODS AND MATERIALS

### 2.1 Animals and surgical procedures

All animals (8‐10 weeks of age) were handled according to the guidelines of the Institutional Animal Care and Use Committee of Soochow University. PTEN^flox/flox^ mice and Advilin‐Cre mice were bred together to generate DRG sensory neuronal specific PTEN knockout mice. Sciatic nerve injury and in vivo electroporation of adult DRG neurons were performed as described previously.^8^

### 2.2 Reagents and antibodies

The Tuj1 mouse mAb (1:1500, MMS‐435P) antibody was purchased from Covance. The PTEN (1:1000, sc‐7974) antibody was purchased from Santa Cruz Biotechnology. Secondary antibodies conjugated with Alexa fluorophores 488 or 568 (1:1000) and Alexa Fluor 488‐conjugated IB4 were from Invitrogen. The SF1670 and BPV were purchased from Selleck.

### 2.3 Neuronal culture and Axon length analysis

Adult sensory neurons were cultured as described previously.^9^ The isolated neurons were allowed to grow axons for 3 days, and we administered SF1670 (1 or 10 nM) or 200 nM BPV to the culture medium. The longest axons of one hundred randomly selected neurons in each experimental condition were measured using AxioVision 4.7 software. Average axon lengths were calculated from three separate experiments in each condition.

### 2.4 Immunofluorescence staining

DRG tissues were cut into slices of 14 microns. The primary antibody was applied for 24 hour at 4°C and the secondary antibodies for 1 hour at room temperature. For immunofluorescence staining of IB4, Alexa Fluor 488‐conjugated IB4 was applied overnight at 4°C and mounted with a MOWIOL media.

### 2.5 Western blots and qRT‐PCR

The protein samples were loaded into SDS‐PAGE gels and then transferred to polyvinylidene fluoride membranes (PVDF). Protein bands were visualized using Pierce™ ECL Western Blotting Substrate. TRIzol reagent was used to extract RNA from the DRG tissue. The PCR products were labelled using the CFX96™ real‐time PCR detection system (Bio‐Rad).

The following primer sequences were used:

PTEN‐F: 5’‐CTCCTCTACTCCATTCTTCCC‐3’

PTEN‐R: 5’‐ACTCCCACCAATGAACAAAC‐3’

### 2.6 Statistics

Data are presented as mean ± SEM. Two‐tailed Student’s t tests were used to compare the different experimental conditions. *P* < 0.05 was considered statistically significant.

## 3 RESULTS

### 3.1 Inhibition of PTEN promotes adult sensory neuronal axon growth

**FIGURE 1:**
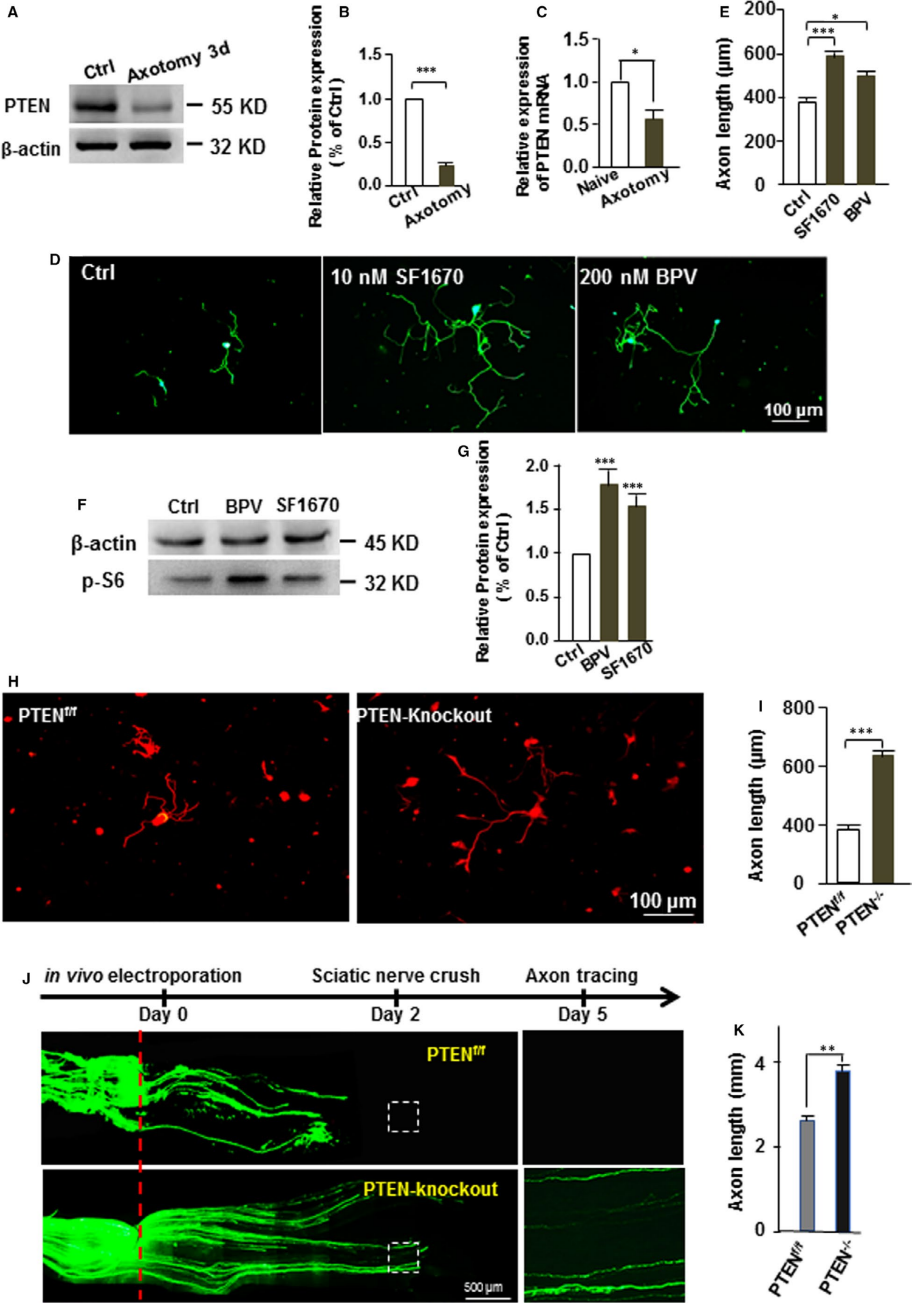
Inhibition of PTEN activity promotes adult sensory neuronal axon growth. (A) Representative Western blot images of PTEN expression in L4‐L5 DRGs of adult mice 3 days after sciatic nerve axotomy. Compared to uninjured control, PTEN protein level is markedly down‐regulated by peripheral axotomy (n = 3). (B) Quantification of Western blot images of PTEN expression. The relative protein level of PTEN in the L4 DRGs was reduced 3 days after sciatic nerve axotomy (n = 3). ***P < 0.001. (C) Quantification of PTEN mRNA levels by qPCR. The relative level of PTEN mRNA expression in the L4 DRGs was reduced 3 days after sciatic nerve axotomy (n = 3). * *P* < 0.05. (D) Adult DRG neurons were cultured in vitro for 3 days and treated with PTEN inhibitor SF1670 (10 nM) or BPV (200 nM). The vehicle DMSO was as control. All neurons were stained with anti‐βIII tubulin (green). Scale bar: 100 μm. Quantification of the average length of the longest axons (n = 3). (E) The pharmacological inhibition of PTEN activity promoted the axon growth of peripheral sensory neurons (n = 3). * *P* < 0.05, *** *P* < 0.001. (F) The pharmacological inhibition of PTEN activity markedly increases phospho‐S6 expression, which is a well‐known downstream target of the PTEN, in the cultured DRG neurons. (G) Quantification of Western blot images of phospho‐S6 expression. The relative protein level of phospho‐S6 was increased in the cultured DRG neurons. (n = 3). *** *P* < 0.001. (H) The adult DRG neurons were cultured from the Advilin‐Cre induced PTEN knockout mice. Scale bar: 100 μm. (I) Axon length of adult DRG neuron in the PTEN knockout mice was significantly longer than the PTEN^flox/flox^ mice (n = 3). ***P < 0.001. (J) The left L3‐L4 DRGs were electroporated with EGFP plasmid, and sciatic nerve was crushed two days after electroporation. Another three days later, whole sciatic nerve segment was harvested. Red dot line is crush site. Arrow head is regenerating axons. Scale bar: 500 μm. (K) PTEN knockout significantly promotes sciatic nerve axon regeneration in vivo (n = 5). *** *P* < 0.001

We found that PTEN expression in DRG neurons was significantly down‐regulated compared to the control after nerve injury Figure 1A‐C. Our data also showed that pharmacological inhibition of PTEN markedly increased phospho‐S6 expression in the cultured DRG neurons and promotes its axon growth Figure 1D‐G). Consistently, the axon length in the PTEN knockout mice was significantly longer than in the wild‐type mice Figure 1H‐I. Furthermore, knockout of PTEN also markedly promoted in vivo sciatic nerve axon regeneration Figure 1J‐K). Taken together, our results indicate that the inhibition of PTEN promotes axon growth in adult sensory neurons in vitro and in vivo.

### 3.2 IB4^+^ neurons have a higher PTEN expression than IB4^‐^ neurons

**FIGURE 2:**
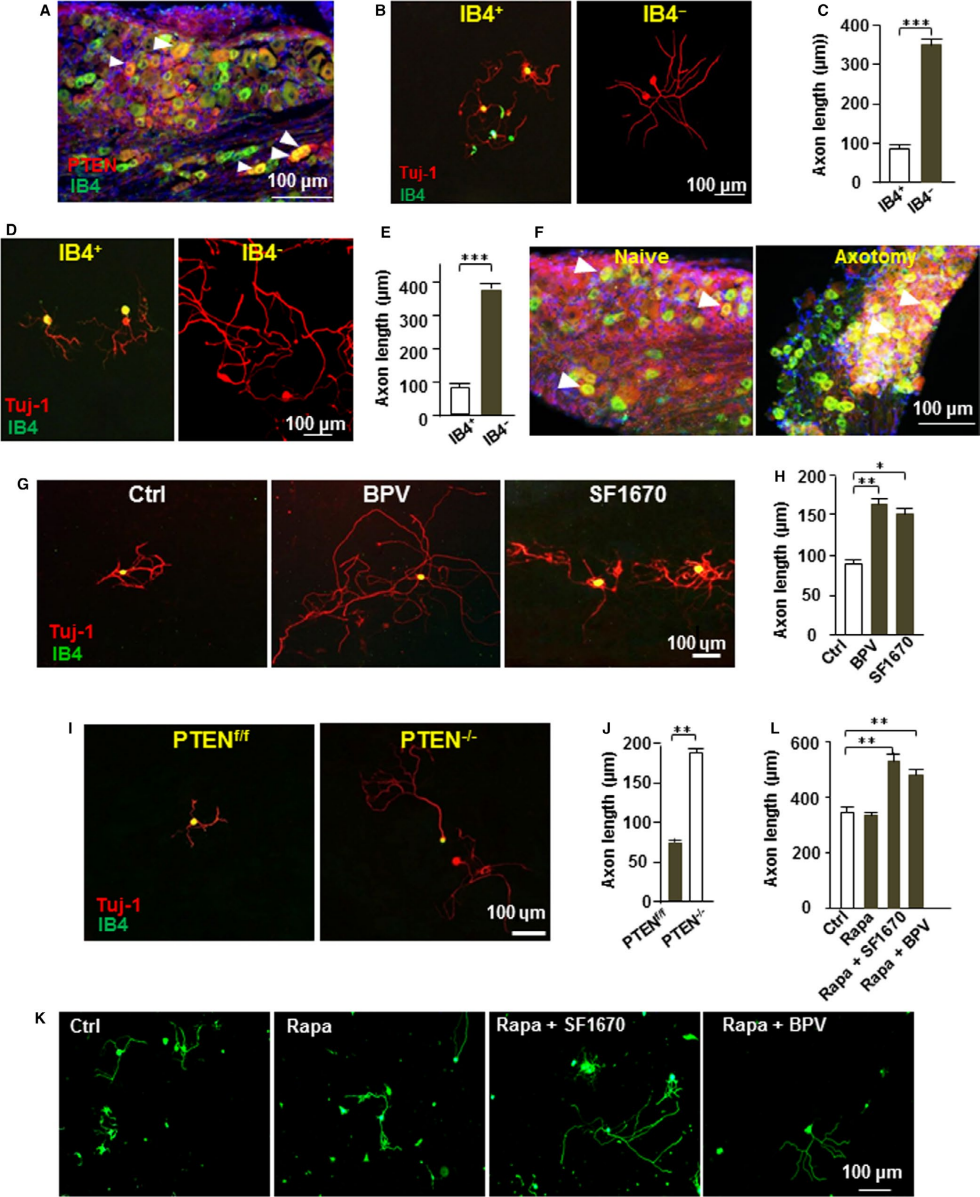
IB4^+^ neurons possess higher PTEN expression, and PTEN inhibition promotes its axon growth independent of mTOR. (A) Immunohistological staining of L4 DRG section showed that IB4+neurons possess higher PTEN expression, and about 36.3 ± 8.1% of DRG neurons showed positive for both PTEN and IB4 staining. Scale bar, 100 μm. (B) Adult DRG neurons were cultured for three days and stained with anti‐βIII tubulin (red) and Alexa Fluor 488‐conjugated Isolectin IB4 (green). Scale bar, 100 μm. (C) Axon length from IB4^+^ neurons was significantly shorter than IB4^‐^ neuron (n = 3). ***P < 0.001. (D) Adult DRG neurons were cultured for 18 hours after peripheral nerve axotomy and stained for anti‐βIII tubulin (red) and Alexa Fluor 488‐conjugated Isolectin IB4 (green). Scale bar, 100 μm. (E) IB4^‐^ neuronal axon growth is significantly enhanced by peripheral axotomy; however, intrinsic axon growth ability of IB4^+^ neuron is not affected (n = 3). ***P < 0.001. (F) Immunohistostaining of L4 DRG section showed that PTEN expression in IB4^+^ neurons was not affected by sciatic nerve axotomy. Arrowhead: IB4^+^ neurons. Scale bar, 100 μm. (G) Adult DRG neurons were cultured for three days with PTEN inhibitor SF1670 (10 nM) or BPV (200 nM) treatment. Vehicle (DMSO) was as control. All neurons were stained with anti‐βIII tubulin (red) and Alexa Fluor 488‐conjugated Isolectin IB4 (green). Scale bar, 100 μm. (H) Administration of PTEN inhibitor significantly increases axon length of IB4 + neuron (n = 3). *P < 0.05, **P < 0.01. (I) Adult DRG sensory neurons were culture for three days from advilin‐cre induced PTEN knockout mice. All neurons were stained with anti‐βIII tubulin (red) and Alexa Fluor 488‐conjugated Isolectin IB4 (green). Scale bar, 100 μm. (J) Quantification of the average axon length showed that PTEN knockout markedly promotes IB4^+^ neuronal axon growth (n = 3). ** *P* < 0.01. (K) Adult DRG neurons were cultured with rapamycin (20 nM) only, or cultured with both rapamycin 20 nM and PTEN inhibitor (10 nM SF1670 or 200 nM BPV). All neurons were stained with anti‐βIII tubulin (green). Scale bar, 100 μm. (L) Quantification of the average axon length showed that mTOR inhibition did not affect the axon growth promoting effect of the PTEN inhibitor (n = 3). **P < 0.01

**O**ur data revealed that PTEN expression in the IB4^+^ neurons was significantly higher than in the IB4^‐^ neurons Figure 2A, and the average axon length of the IB4^+^ neurons was significantly shorter than the IB4^‐^ neurons Figure 2B and C. It has been demonstrated that peripheral axotomy can activate the intrinsic axon regeneration ability of adult sensory neurons.^10^ Although peripheral axotomy significantly promotes axon growth in IB4^‐^ neurons Figure 2D and E, however, we found that peripheral axotomy did not affect the PTEN expression in the IB4^+^ neurons Figure 2F, and axon growth from the IB4^+^ neurons was also not enhanced by peripheral axotomy Figure 2D and E. These results suggested that PTEN protein is a potential inhibitor of the IB4^+^ neuronal axon growth.

### 3.3 Inhibition of PTEN promotes axon growth in IB4^+^ neurons independent of mTOR

Next, we found that the axon length of IB4^+^ neurons was longer in the SF1670 (10 nM) and BPV (10 nM) treated groups Figure 2G and H). Consistently, the axon length of the IB4^+^ neurons from the PTEN knockout mice was also increased compared to the control mice Figure 2A and B. Then, DRG neurons were cultured for 3 days with both PTEN and mTOR inhibitors. Our data indicated that mTOR inhibition did not affect the axon growth promoting effect of the PTEN inhibitor Figure 2I and J). This means that the regulatory effect of PTEN on adult sensory neurons is not dependent on the mTOR pathway.

## 4 DISCUSSION

Although the damaged peripheral neurons can regenerate axons, however, accumulating evidence has shown that still some parts of adult sensory neurons cannot regenerate axons after injury, such as IB4^+^ neurons^7^, ^11^. In agreement with these previous findings, our immunohistological staining revealed that the expression of PTEN in IB4^+^ neurons was significantly higher than in IB4^‐^ neurons. Here, our data further showed that the inhibition of PTEN markedly promotes IB4^+^ neuronal axon regeneration. Thus, it means that PTEN is a critical repressor of IB4^+^ neuronal axon regeneration.

It has been reported that PTEN regulates mature CNS axon regeneration through the activation of the mTOR pathway^12^. However, our data showed that the mTOR inhibitor did not affect the axon growth promoting effect of PTEN deletion in sensory neurons. Therefore, the results suggest that PTEN regulates sensory neuronal axon growth independently of the mTOR pathway. Glycogen synthase kinase‐3 beta (GSK3β) is well‐known downstream substrate of PTEN‐Akt pathway. An earlier study conducted by our team showed that the inactivation of GSK3β is necessary for sensory axon regeneration^9^. Thus, it is possible that PTEN controls sensory axon regeneration through GSK3β.

Summary, our results demonstrated that IB4^+^ sensory neurons express a high level of PTEN and that PTEN inhibition dramatically promotes axon growth of both IB4^+^ sensory neurons and IB4^‐^ neurons. Taken together, these data indicate that PTEN is a prominent repressor of adult sensory axon regeneration, especially in IB4^+^ neurons.

## CONFLICT OF INTEREST

The authors confirm that there are no conflicts of interest.

## AUTHORS' CONTRIBUTIONS

LY Z, F H, SB Q, JJ M, YX M, HC Z, JL X, XY F, JQ C, HL Y, F Z, and Saijilafu designed the experiment. LY Z, F H and SB Q performed the experiments. LY Z, F Hand Saijilafu co‐wrote the manuscript. Liyu Zhou: Investigation (equal); Writing‐original draft (equal). Feng Han: Investigation (equal); Writing‐original draft (equal). Shibin Qi: Investigation (equal). Jinjin Ma: Data curation (supporting). Yanxia Ma: Data curation (supporting). Jile Xie: Formal analysis (supporting). Hongcheng Zhang: Methodology (supporting). Xinya Fu: Formal analysis (supporting). Jianquan Chen: Supervision (supporting). Bin Li: Supervision (supporting). Huilin Yang: Supervision (equal); Validation (equal). Feng Zhou: Supervision (equal); Validation (equal). Saijilafu N/A: Supervision (equal); Validation (equal); Writing‐review & editing (equal).

## Data Availability

The data that support the findings of this study are available from the corresponding author upon reasonable request.
